# Group cognitive behavioural therapy and weight regain after diet in type 2 diabetes: results from the randomised controlled POWER trial

**DOI:** 10.1007/s00125-017-4531-9

**Published:** 2018-01-09

**Authors:** Kirsten A. Berk, Hanneke I. M. Buijks, Adrie J. M. Verhoeven, Monique T. Mulder, Behiye Özcan, Adriaan van ’t Spijker, Reinier Timman, Jan J. Busschbach, Eric J. Sijbrands

**Affiliations:** 1 Section of Pharmacology, Vascular and Metabolic Diseases Section, Department of Internal Medicine, Erasmus MC – Office D435, ’s Gravendijkwal 230, PO-Box 2040, 3000 CA Rotterdam, the Netherlands; 2 Department of Psychiatry, Section of Medical Psychology and Psychotherapy, Erasmus MC, Rotterdam, the Netherlands

**Keywords:** Cognitive behavioural therapy, Diabetes mellitus type 2, Obesity, Psychological intervention, Very low calorie diet, Weight maintenance

## Abstract

**Aims/hypothesis:**

Weight-loss programmes for adults with type 2 diabetes are less effective in the long term owing to regain of weight. Our aim was to determine the 2 year effectiveness of a cognitive behavioural group therapy (group-CBT) programme in weight maintenance after diet-induced weight loss in overweight and obese adults with type 2 diabetes, using a randomised, parallel, non-blinded, pragmatic study design.

**Methods:**

We included 158 obese adults (median BMI 36.3 [IQR 32.5–40.0] kg/m^2^) with type 2 diabetes from the outpatient diabetes clinic of Erasmus MC, the Netherlands, who achieved ≥5% weight loss on an 8 week very low calorie diet. Participants were randomised (stratified by weight loss) to usual care or usual care plus group-CBT (17 group sessions). The primary outcomes were the between-group differences after 2 years in: (1) body weight; and (2) weight regain. Secondary outcomes were HbA_1c_ levels, insulin dose, plasma lipid levels, depression, anxiety, self-esteem, quality of life, fatigue, physical activity, eating disorders and related cognitions. Data were analysed using linear mixed modelling.

**Results:**

During the initial 8 week dieting phase, the control group (*n* = 75) lost a mean of 10.0 (95% CI 9.1, 10.9) kg and the intervention group (*n* = 83) lost 9.2 (95% CI 8.4, 10.0) kg (*p* = 0.206 for the between-group difference). During 2 years of follow-up, mean weight regain was 4.7 (95% CI 3.0, 6.3) kg for the control group and 4.0 (95% CI 2.3, 5.6) kg for the intervention group, with a between-group difference of −0.7 (95% CI −3.1, 1.6) kg (*p* = 0.6). The mean difference in body weight at 2 years was −1.2 (95% CI −7.7, 5.3) kg (*p* = 0.7). None of the secondary outcomes differed between the two groups.

**Conclusions/interpretation:**

Despite increased treatment contact, a group-CBT programme for long-term weight maintenance after an initial ≥5% weight loss from dieting in obese individuals with type 2 diabetes was not superior to usual care alone.

**Trial registration:**

Trialregister.nl NTR2264

**Funding:**

The study was funded by the Erasmus MC funding programme ‘Zorgonderzoek’ (grant 2008-8303).

**Electronic supplementary material:**

The online version of this article (10.1007/s00125-017-4531-9) contains peer-reviewed but unedited supplementary material, which is available to authorised users.


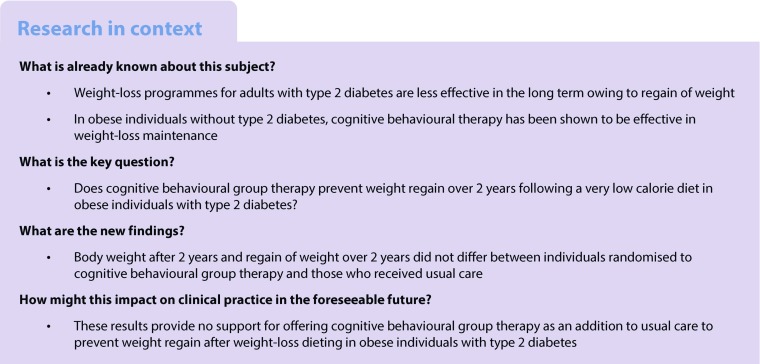


## Introduction

Lifestyle interventions that reduce weight improve a number of cardiovascular disease risk factors in adults with type 2 diabetes [1, 2], but studies using hard endpoints have been disappointing. In the Look Action for Health in Diabetes (AHEAD) trial, an intensive lifestyle intervention led to a number of beneficial effects on health and quality of life, but not on cardiovascular outcomes [3]. Weight regain during follow-up in a substantial proportion of the participants may have contributed to this unexpected outcome [4, 5]. Obviously, non-invasive weight-loss interventions require strategies that prevent regain of body weight to achieve clinically relevant effects.

Cognitive behavioural therapy (CBT) in general, and cognitive behavioural group therapy (group-CBT) in particular, has been shown to be effective in weight management and weight-loss maintenance after dieting in obese adults without type 2 diabetes [6–9]. While standard behavioural therapy focuses on techniques to change behaviour directly (e.g. self-monitoring, goal setting), CBT aims to change dysfunctional thoughts about self-image and behaviour into more realistic, helpful thoughts that may facilitate long-term behavioural change. Obese people with type 2 diabetes differ from the ‘healthy’ obese in many ways: they have more metabolic problems, greater use of medication, higher prevalence of comorbidities, and lower quality of life, all of which may potentially affect an individual’s ability to lose or maintain weight. In obese individuals with type 2 diabetes, lifestyle interventions using standard behavioural therapy have shown only a moderate effect on weight loss [10], while the two observational studies published to date on the effect of group-CBT as part of weight-loss programmes have shown more favourable effects on weight loss [11, 12]. However, the effect of group-CBT on weight-loss maintenance has not been investigated in individuals with type 2 diabetes.

The primary objective of this study was to determine the 2-year effectiveness of a group-CBT programme on top of usual care in weight maintenance after diet-induced weight loss in overweight and obese adults with type 2 diabetes, using a pragmatic study design. Secondary objectives were to evaluate the effects of group-CBT on cardiovascular risk factors and psychological wellbeing.

## Methods

### Study design

The protocol of the ‘Prevention of Weight Regain’ (POWER) trial has been published previously [13]. The POWER study was a parallel-group, randomised controlled trial that was conducted between March 2010 and May 2015. The study was approved by the medical ethics committee of the Erasmus MC (MEC-2009-143/NL26508.078.09) in compliance with the Helsinki Declaration of 2008. All participants provided written informed consent.

### Study population

Overweight and obese (BMI >27 kg/m^2^) adults with type 2 diabetes and aged 18–75 years were recruited from the outpatient diabetes clinic of Erasmus MC. This hospital is a tertiary referral centre, but individuals with severe comorbidities were excluded from this trial. Some of the participants were referred by their general practitioners specifically for participation in this trial. Exclusion criteria were: pregnancy; lactation; inadequate understanding of the Dutch language; severe psychiatric problems; significant cardiac arrhythmias; unstable angina; decompensated congestive heart failure; carcinomas; major organ system failure; untreated hypothyroidism; end-stage renal disease; and myocardial infarction, cerebrovascular accident or major surgery during the previous 3 months. We recorded the age, sex and ethnicity of individuals who met the eligibility criteria but declined to participate.

### Weight-loss dieting

After collection of baseline data, participants started with a diet very low in energy (very low calorie diet; VLCD) of approximately 3140 kJ (750 kcal)/day for 8 weeks. Blocks of 20 participants started with the diet concomitantly. The daily diet consisted of two diabetes-specific meal replacements (Glucerna, Abbott Nutrition, Columbus, OH, USA) plus 75 g lean meat, 150 ml skimmed milk and low-carbohydrate vegetables ad libitum. To reduce the risk of hypoglycaemia, doses of sulfonylurea derivatives and insulin were reduced at the start of the dietary intervention.

After 8 weeks the diet was changed into a low-energy diet of 4600–5400 kJ (1100–1300 kcal)/day, gradually increasing the intake during the following 12 weeks. From then on, the participants ate a diet based on national health recommendations, aiming at weight maintenance. During the entire study, 60 min of moderately intensive exercise each day was recommended, and glucose-lowering medication and insulin doses were adjusted by the responsible physician based on plasma glucose levels. Other medications remained unchanged.

### Randomisation and masking

After 8 weeks of the VLCD, participants who had lost ≥5% of their body weight were randomly assigned to either the control group or the intervention group with an allocation ratio of 1:1. The block randomisation was stratified by weight loss, with categories 5–7.5%, 7.5–10% and >10%. The participants, CBT therapists and primary researcher (K. Berk) were not blinded to the intervention, whereas the medical team at the outpatient clinic was. Participants were not allowed to talk to their medical team about the group-CBT sessions. The treatment of diabetes and its complications (according to national guidelines) was not influenced by the allocation of the participants. Blinded medical assistants took measurements (i.e. weight, waist circumference, blood pressure) and all statistical analyses were independently conducted by two researchers.

### Control group: usual care

The control group received the usual care for diabetes regulation and cardiovascular risk management at our tertiary medical referral centre. This consisted of scheduled visits every 3–6 months (sometimes on separate occasions) to the internist and diabetes nurse, plus referral to a dietitian or psychologist when indicated. In addition, during the diet period participants using insulin frequently contacted the diabetes nurse by email or telephone to optimally adjust their insulin dose according to their glucose levels. The increased attention (during additional visits) given to the intervention group was not compensated for in the control group.

### Intervention group: usual care plus group-CBT

After randomisation, participants allocated to the intervention group started group-CBT with up to ten participants per group. The first ten weekly sessions were followed by two fortnightly sessions, two monthly sessions and two 3-monthly sessions, with the last session taking place 18 months after randomisation. The group-CBT sessions were conducted by a trained psychologist/psychotherapist (H. Buijks or A. van ’t Spijker) experienced in CBT as well as in diabetes care.

The protocol of the first ten group-CBT sessions was based on the cognitive therapy described by Werrij et al [7]. The aim of the first ten sessions, including one partner session, was to restructure dysfunctional cognitions on lifestyle, weight and body perception. The last seven sessions were devoted to challenging dysfunctional cognitions of relapse. Only when participants had fully mastered the cognitive behavioural techniques were proactive coping and problem-solving techniques [14–16] explained. A detailed description of the intervention has been published previously [13]. Group-CBT was given in addition to usual care.

### Outcome measures

All primary and secondary outcome variables were prespecified and are described in more detail in the study protocol [13]. Outcome variables were assessed at baseline, at randomisation after 8 weeks of VLCD (primary outcome only), at 12 weeks (after the weekly group-CBT sessions had finished) and at 52, 78 and 104 weeks after randomisation.

The primary endpoints were the difference between the study groups in body weight (kg) after 2 years of follow-up and in weight regain (kg) from randomisation to 2 years. Weight was measured to the nearest 0.1 kg after removal of shoes, using a Seca 888 compact digital flat scale (Seca, Hamburg, Germany).

Secondary outcomes (all defined as between-group differences) were as follows: change in weight (kg) from baseline to 2 years of follow-up; 2 year estimates and change from baseline to 2 years in waist circumference (cm); systolic blood pressure (mmHg); total cholesterol (mmol/l), LDL-cholesterol (mmol/l), HDL-cholesterol (mmol/l), triacylglycerol (mmol/l) and HbA_1c_ (% and mmol/mol), all measured via routine laboratory techniques; insulin dose (U/day); depression and anxiety (Hospital Anxiety and Depression Scale [HADS] [17, 18]); self-esteem (Rosenberg Self-Esteem Scale [19]); quality of life (EuroQol five-dimension questionnaire [20, 21]); fatigue (Checklist Individual Strength [22, 23]); physical activity (Short Questionnaire to Assess Health Enhancing Physical Activity [24]); and eating disorders (Eating Disorder Examination Questionnaire [EDE-Q] [25]) and related cognitions (EDE-Q subscores of eating restraint, eating concern, weight concern and shape concern; score range 0–6). In addition to the predefined outcome variables, we recorded the number of visits to physicians and paramedics at the diabetes outpatient clinic. We counted visits to each specialist separately, even when they occurred on the same day. We also counted contacts by email or phone (as 0.333 of a visit). We managed our data using the trial management system OpenClinica (Waltham, MA, USA).

### Sample size

To base the sample-size calculation on realistic 8-week weight-loss data, an independent statistician carried out a blinded power calculation after the first 75 participants had completed the 8-week VLCD period, as described in the protocol [13]. Sample size was calculated with SPSS version 21.0 (www-01.ibm.com/support/docview.wss?uid=swg21608060) using the mixed-model ANOVA procedure described by Aberson [26]. Alpha was set at 0.05, power at 0.80 and the baseline–end correlation at 0.90. A clinically relevant difference between the treatment groups was set at 5% weight loss [27]. This calculation yielded a requirement for 52 participants in each group. Anticipating a dropout rate of 25%, we aimed for a total sample size of 140. We also conducted a post hoc power calculation. With an *α* of 0.05 and true baseline–end correlation of 0.94, the power was 0.998 for the intention-to-treat analysis and 0.969 for the per-protocol analysis.

### Statistical analyses

Normality of the data and homogeneity of variances were tested using the Shapiro–Wilks test and Levene’s test. Variables are expressed as numbers with percentages, means with SDs, medians with interquartile ranges or means with 95% CI. Differences between the control and intervention groups at baseline were tested using a *χ*^2^ test, an independent samples *t* test or a Mann–Whitney *U* test, depending on the normality of the data. Linear mixed modelling was applied for analyses of between-group differences for the 2-year course for the primary and secondary outcomes. This method efficiently handles data with missing and unbalanced time points, and corrects for selective dropout when the missing values are dependent on variables present in the model (missing at random) [28].

The models included three levels: group membership (the highest level), participants (intermediate level) and their repeated measures (lower level). The need for the upper (group membership) level was determined with the deviance statistic [29]. The fixed parts of the models included the allocation arm; sex; linear, quadratic and logarithmic time effects; and the interactions of allocation and sex with time effects. Variance components matrices were applied for the covariance structures. All analyses were conducted according to the intention-to-treat and per-protocol principles. Treatment was considered per-protocol when participants attended at least nine group-CBT sessions [13]. Per-protocol analyses were restricted to the control group and compliant participants in the intervention group. The difference in the number of visits to the outpatient clinic during the study was analysed using the Mann–Whitney *U* test. Results with *p* values of <0.05 were considered statistically significant. Analyses were carried out using SPSS version 21.0.

## Results

**Fig. 1:**
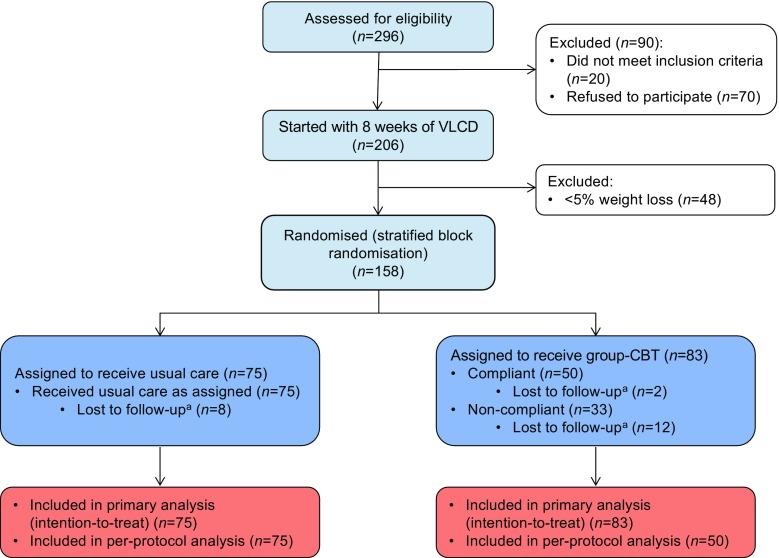
Study flow-chart. ^a^Reasons for loss to follow-up: three individuals became pregnant, two moved to another country, two moved to another hospital, three underwent bariatric surgery, two were diagnosed with carcinoma, five no longer wanted to participate and five failed to respond to invitations for follow-up visits

Of the 296 individuals who were assessed from March 2010 until May 2013, 276 were eligible to participate in our study and 206 gave written informed consent (Fig. 1). Compared with individuals who participated in the study, those who declined to participate were older (56.1 ± 10.3 vs 53.0 ± 10.8 years, *p* = 0.05) and more often male (61% vs 43%, *p* = 0.008). The main reasons for refusal to participate were related to work and a lack of time.

After the 8-week VLCD, 158 participants (77%; median baseline BMI 36.3 [IQR 32.5–40.0] kg/m^2^) had lost ≥5% of their body weight and were randomised to either the intervention arm (*n* = 83) or the control group (*n* = 75). The difference in numbers between the study arms resulted from the block randomisation stratified by weight loss. The baseline characteristics of participants who did and did not achieve ≥5% weight loss are compared in the electronic supplementary material (ESM) Table 1. The excluded individuals had significantly higher baseline HbA_1c_ levels, at 69.5 (59.3–86.3) mmol/mol (8.5% [7.6–10.0%]) vs 60.0 (53.0–69.0) mmol/mol (7.6% [7.0–8.5%]) (*p* = 0.001), and a higher anxiety score (8.0 [4.0–11.0] vs 6.0 [3.0–9.0], *p* = 0.038) than the included participants (all median [IQR]).

Baseline characteristics did not significantly differ between the control and intervention groups (Table 1), except for the EDE-Q score (*p* = 0.021). Eight participants in the control group and 14 in the intervention group were lost to follow-up at different time points during the study (*p* = 0.358) (Fig. 1). Those participants were kept in the analyses as data with missing time points.

**Table 1:** Baseline participant characteristics

**Characteristic**	**Control (*n*** **= 75)**	**Intervention (*n*** **= 83)**
Age (years)	55.2 ± 9.3	52.3 ± 11.3
Age range (years)	32–73	28–74
Female	44 (58.7)	44 (53.0)
European descent	45 (60.0)	42 (50.6)
Low education	25 (33.3)	21 (25.3)
Employed	24 (32.0)	36 (43.4)
Employment (days/week)	0 (0–3.3)	0 (0–5)
Time from type 2 diabetes diagnosis (years)	10.0 (3.0–15.0)	8.0 (3.5–16.0)
Weight (kg)	106.7 ± 22.5	105.5 ± 19.3
BMI (kg/m^2^)	35.7 (32.9–40.9)	36.7 (31.7–39.4)
Waist circumference (cm)	120.4 ± 12.9	119.4 ± 14.2
Systolic blood pressure (mmHg)	145.0 ± 20.9	138.6 ± 18.6
Diastolic blood pressure (mmHg)	81.0 ± 10.5	80.2 ± 10.7
HbA_1c_ (mmol/mol)	61.0 (53.8–68.3)	58.0 (51.5–72.0)
HbA_1c_ (%)	7.7 (7.1–8.4)	7.5 (6.9–8.7)
Fasting glucose (mmol/l)	8.7 (6.9–10.5)	8.2 (6.8–10.8)
Total cholesterol (mmol/l)	4.4 (3.7–5.1)	4.5 (3.9–5.2)
LDL-cholesterol (mmol/l)	2.5 (2.0–3.0)	2.6 (2.2–3.1)
HDL-cholesterol (mmol/l)	1.2 (1.0–1.4)	1.1 (1.0 − 1.3)
Triacylglycerol (mmol/l)	1.7 (1.3–2.4)	2.0 (1.3–2.7)
Insulin users	49 (65.3)	52 (62.7)
Insulin dose among users (U/day)	100.1 ± 42.2	95.7 ± 54.9
Statin users	50 (66.7)	59 (71.1)
Clinical depression	12 (16.0)^a^	15 (18.1)^a^
Clinical anxiety disorder	12 (16.0)^a^	16 (19.3)^a^
Self-esteem (RSE score)	32.0 (28.0–35.0)	32.5 (27.0–35.0)
Quality of life (EQ-5D score)	0.78 (0.57–0.84)	0.81 (0.65–1.0)
Fatigue (CIS subscore 1)	37.0 (27.3–47.8)	36.5 (28.0–47.8)
Eating disorder (EDE-Q score)	1.9 ± 1.0	2.4 ± 1.2
Physical activity (SQUASH score)	2350 (1260–5355)	3495 (1440–5978)

Data are mean ± SD, median (interquartile range) or *n* (%)

^a^Per cent with HADS score >10

CIS, Checklist Individual Strength; EQ-5D, EuroQol five-dimension questionnaire; RSE, Rosenberg Self-Esteem Scale; SQUASH, Short Questionnaire to Assess Health Enhancing Physical Activity

The average number of usual care visits to the outpatient diabetes clinic during the 2 years of follow-up was similar in the intervention and control groups, at 12.0 (8.0–15.0) vs 13.0 (8.0–17.0) visits, respectively (*p* = 0.495). In addition, participants in the intervention group attended a median of 9.0 (5.0–14.0) group-CBT sessions. A total of 33 participants missed more than eight sessions and were considered non-compliant. Non-compliant participants attended a median of 4.0 (0.0–7.0) group-CBT sessions, while compliant participants attended 14.0 (11.0–15.3) sessions. Non-compliant participants were significantly younger than compliant participants (49.0 [38.5–55.5] vs 56.0 [49.5–63.0] years; *p* = 0.004). The main reasons mentioned for non-compliance were health problems and lack of time (work related). Overall, 20% of participants in the intervention group and 12% in the control group reported consulting an external psychologist (*p* = 0.187), with a median number of 3.5 (2.0–5.8) visits in the intervention group and 4.0 (1.0–8.5) visits in the control group over 2 years (*p* = 0.978). Overall, 61% of participants in the intervention group and 63% in the control group were referred to the diabetes team’s dietitian as part of usual care (*p* = 0.218), with a median of 2.0 (1.0–4.8) and 1.0 (0–3.0) visits during the 2 years of follow-up, respectively (*p* = 0.082).

### Weight change during the trial

During the initial 8 weeks of dieting, a mean weight loss of 10.0 (95% CI 9.1, 10.9) kg was observed in the control group and 9.2 (95% CI 8.4, 10.0) kg in the intervention group (*p* = 0.206 for between-group difference). At 2 years of follow-up, mean weight loss was 5.3 (95% CI 3.5, 7.2) kg and 5.2 (95% CI 3.4, 7.1) kg, respectively (*p* = 0.951 for between-group difference). Overall, 38.6% of participants still had weight loss of ≥5% after 2 years of follow-up, including 17.7% whose weight loss remained ≥10%. During the 2 year follow-up, 19.0% of participants managed to fully maintain their lost weight. These percentages were similar in both study arms (*χ*^2^ = 0.161, *p* = 0.688; *χ*^2^ = 0.307, *p* = 0.580; and *χ*^2^ = 0.077, *p* = 0.781, for participants who maintained ≥5%, ≥10% and complete weight loss, respectively).

### Primary outcome

We did not find a significant difference in mean body weight between the intervention and control groups at 2 years of follow-up (intention-to-treat analysis; Table 2), at which point the mean between-group difference was −1.2 (95% CI −7.7, 5.3) kg (*p* = 0.717). In the per-protocol analysis the between-group difference was −3.8 (95% CI −11.5, 3.8) kg (*p* = 0.323). Mean weight regain during follow-up was 4.7 (95% CI 3.0, 6.3) kg for the control group and 4.0 (95% CI 2.3, 5.6) kg for the intervention group at 2 years (46.7% and 43.0% of the lost weight at randomisation, respectively; Table 2), with a between-group difference of −0.7 (95% CI −3.1, 1.6) kg (*p* = 0.556) in the intention-to-treat analysis and −0.6 (95% CI −3.3, 2.0) kg (*p* = 0.635) in the per-protocol analysis. The deviance statistic of the linear mixed model indicated that a three-level model with a third upper ‘group’ level was not significantly better than a two-level model with time and allocation (*χ*^2^_(1)_ = 1.189; *p* = 0.28). ESM Table 2 shows the estimates of the linear mixed model for weight during the study (intention-to-treat). These results indicate that there was no allocation effect and no allocation–time interaction. We also found no interaction of sex with time and allocation (data not shown).

**Table 2:** Weight and weight regain over 2 years of follow-up

**Outcome**	**Control group**	**Intervention group**	**Between-group difference**
Weight (kg)			
−8 weeks (baseline)	106.8 (102.3, 111.2)	105.5 (101.3, 109.7)	−1.3 (−7.4, 4.9)
0 weeks (randomisation)	96.8 (92.4, 101.2)	96.3 (92.1, 100.5)	−0.5 (−6.6, 5.6)
12 weeks	95.7 (91.3, 100.1)	95.2 (91.0, 99.4)	−0.4 (−6.5, 5.7)
52 weeks	98.5 (94.0, 103.0)	97.7 (93.4, 102.0)	−0.8 (−7.0, 5.4)
78 weeks	100.6 (96.1, 105.2)	99.6 (95.2, 103.9)	−1.0 (−7.3, 5.3)
104 weeks	101.4 (96.7, 106.1)	100.2 (95.8, 104.7)	−1.2 (−7.7, 5.3)
Weight regain from randomisation (kg)			
52 weeks	1.7 (0.6, 2.8)	1.4 (0.4, 2.5)	−0.3 (−1.8, 1.2)
78 weeks	3.8 (2.5, 5.2)	3.3 (2.0, 4.6)	−0.6 (−2.5, 1.4)
104 weeks	4.7 (3.0, 6.3)	4.0 (2.3, 5.6)	−0.7 (−3.1, 1.6)

Data are means (95% CI)

### Secondary outcomes

**Fig. 2:**
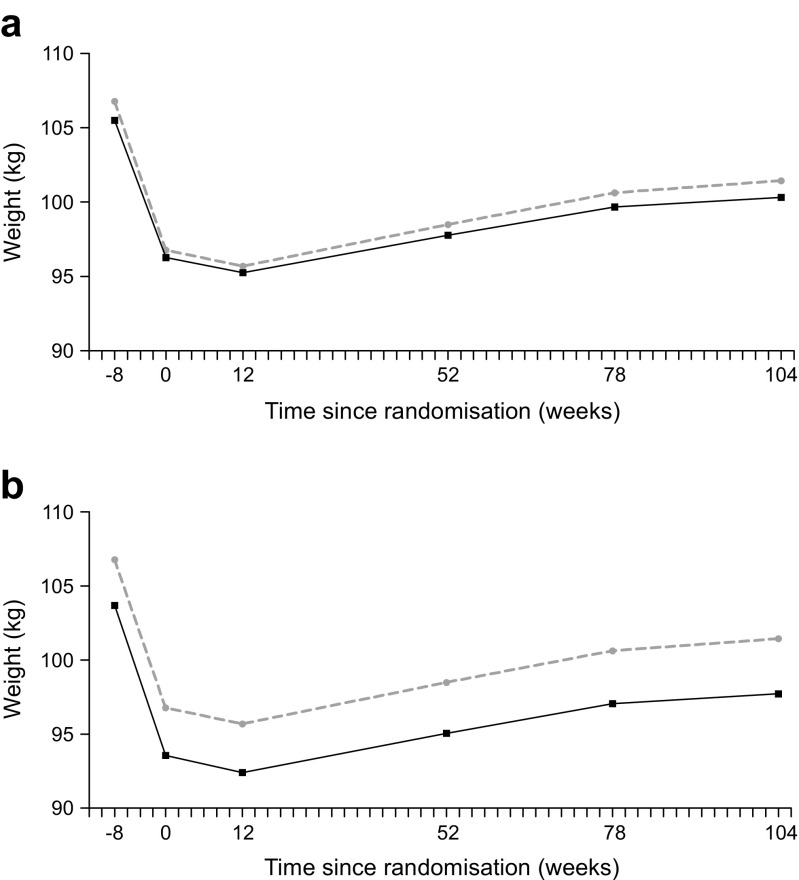
Estimates of weight from baseline to 2 years for the group-CBT and control groups. (**a**) Intention-to-treat and (**b**) per-protocol analysis. Solid line, group-CBT; dashed line, control group. *p* = 0.951 (intention-to-treat) and *p* = 0.613 (per protocol) for between-group difference in course of weight from baseline to 2 years of follow-up, analysed using a mixed-modelling procedure

Change in weight from baseline to 2 years was not significantly different between the two groups, at 0.1 (95% CI −2.5, 2.7) kg (*p* = 0.951) in the intention-to-treat analysis (Fig. 2a) and −0.8 (95% CI −3.7, 2.2) kg (*p* = 0.613) in the per-protocol analysis (Fig. 2b).

None of the other secondary outcomes was significantly different between the intervention and control groups at 2 years (Table 3). Furthermore, the change from baseline was not different between the two groups for any of the secondary outcome variables (data not shown). None of the primary or secondary outcomes differed between the two therapists who conducted the group-CBT (data not shown). At 2 years of follow-up, both the intervention and control groups had a significantly lower mean waist circumference, insulin dose, depression score and fatigue score than at baseline (*p* < 0.05). In addition, the EDE-Q subscale scores on weight and shape concern significantly improved during the 2 years of follow-up for both groups (*p* < 0.01).

**Table 3:** Differences in secondary outcome variables after 2 years of follow-up between the intervention and control groups

**Outcome variables** ^**a**^	**Control group**	**Intervention group**	**Between-group difference**
Waist circumference (cm)	116.0 (112.7, 119.2)	115.0 (111.9, 118.1)	−1.0 (−5.4, 3.5)
Systolic blood pressure (mmHg)	140.0 (135.4, 144.7)	139.2 (134.8, 143.7)	−0.8 (−7.3, 5.6)
HbA_1c_ (mmol/mol)	64.9 (60.6, 69.1)	64.0 (59.8, 68.1)	−0.9 (−6.8, 5.0)
HbA_1c_ (%)	8.1 (7.7, 8.5)	8.0 (7.6, 8.4)	−0.1 (−0.6, 0.5)
Insulin dose (U/day)	43.5 (26.9, 60.1)	40.4 (24.3, 56.6)	−3.1 (−26.3, 20.2)
Total cholesterol (mmol/l)	4.20 (3.90, 4.50)	4.48 (4.19, 4.77)	0.29 (−0.13, 0.70)
LDL-cholesterol (mmol/l)	2.34 (2.10, 2.58)	2.65 (2.42, 2.88)	0.31 (−0.03, 0.64)
HDL-cholesterol (mmol/l)	1.24 (1.16, 1.32)	1.22 (1.14, 1.30)	−0.02 (−0.13, 0.10)
Triacylglycerol (mmol/l)	2.08 (1.50, 2.66)	2.29 (1.73, 2.84)	0.21 (−0.60, 1.01)
Depression (HADS score)	5.3 (4.2, 6.3)	5.5 (4.5, 6.6)	0.3 (−1.2, 1.8)
Anxiety (HADS score)	5.3 (4.1, 6.4)	6.1 (4.9, 7.2)	0.8 (−0.8, 2.4)
Self-esteem (RSE score)	31.1 (29.1, 33.1)	29.9 (27.9, 31.9)	−1.2 (−4.0, 1.7)
Quality of life (EQ-5D score)	0.69 (0.62, 0.76)	0.69 (0.63, 0.76)	0.01 (−0.09, 0.10)
Fatigue (CIS score)	31.2 (27.9, 34.5)	33.4 (30.2, 36.7)	2.2 (−2.4, 6.9)
Eating disorders (EDE-Q score)	1.70 (1.39, 2.01)	2.11 (1.80, 2.41)	0.41 (−0.02, 0.84)
Physical activity (SQUASH score)	4176 (2160, 6191)	5453 (3427, 7480)	1278 (−1580, 4136)

^a^Estimates after 2 years of follow-up and between-group difference (95% CI), analysed via a linear mixed-model procedure according to the intention-to-treat principle

CIS, Checklist Individual Strength; EQ-5D, EuroQol five-dimension questionnaire; RSE, Rosenberg Self-Esteem Scale; SQUASH, Short Questionnaire to Assess Health Enhancing Physical Activity

## Discussion

In this randomised controlled trial, group-CBT did not reduce the problem of weight regain following a successful diet-induced weight reduction in overweight and obese adults with type 2 diabetes. Moreover, the secondary outcomes were not different between the intervention and control groups. For both groups, the average waist circumference, insulin dose, depression score and fatigue score remained significantly lower during follow-up after the VLCD.

Weight regain usually occurs in the first year after weight loss [4, 30–32]. In our study, participants in both groups showed a gradual regain of weight of 43–47% of the initially lost weight at 2 years of follow-up. In the Look AHEAD trial, weight regain ranged from 40% to 60% during the 2 years after achieving maximal weight loss, depending on the level of weight loss achieved after 2 months of lifestyle intervention and despite continued use of one daily meal replacement [33]. In post hoc analyses of the Look AHEAD trial, the subgroup of participants who had lost ≥10% of their initial body weight at 1 year had greater odds of maintaining 10% weight loss at 8 years [4], and experienced a 20% decrease in the incidence of cardiovascular disease compared with the control group [5]. This suggests that substantial sustained weight loss may be beneficial for obese individuals with type 2 diabetes. In our trial, only 18% of participants maintained a weight loss of ≥10% in both the intervention and control groups. Clearly, group-CBT did not improve the magnitude of the sustained weight loss. In the Look AHEAD trial [4], 17% of participants in the control group and 27% of those in the intervention group maintained a weight loss of ≥10% after 8 years of follow-up. These results are more impressive than ours, potentially because of a more intensive intervention or the increase in physical activity during follow-up. Physical activity has been shown to produce small but significant benefits to the maintenance of weight loss [34]. In our study, physical activity did not differ between the groups during the 2 years of follow-up.

VLCDs result in a substantial initial weight loss, and are recommended by the American Diabetes Association for weight loss in obese people with type 2 diabetes [35]. This large initial weight loss creates a good starting situation to test the effectiveness of strategies aiming at long-term weight maintenance. Recent studies using other structured programmes have found limited efficacy in weight maintenance after initial VLCD-induced weight loss, where the prolonged use of meal replacements and high-protein diets holds most promise [36]. The data from these and our study indicate that a multifaceted, long-term support programme is needed to prevent weight regain after VLCD-induced weight loss.

Observational studies specifically reporting on CBT in combination with weight-loss dieting with or without increasing physical activity have shown favourable effects on long-term weight loss in obese individuals with type 2 diabetes [11, 12]. However, since group-CBT was part of an intensive, combined intervention including diet and exercise, no conclusions can be drawn on the effectiveness of group-CBT itself. Clearly, our randomised controlled trial does not support a beneficial effect of group-CBT on top of usual care.

In obese adults without type 2 diabetes, positive effects of group-CBT on weight loss and weight-loss maintenance have been described, and this treatment option has been incorporated into international obesity guidelines [6, 7, 37, 38]. In addition to methodological considerations, our intervention differs from that investigated in these previous studies by having a substantially longer follow-up and restricting participation to individuals with diabetes. Our intervention was based on the protocol of Werrij et al [7]. They found that in obese non-diabetic individuals, group-CBT was superior to increasing physical activity in maintaining diet-induced weight loss. Notably, both the control and intervention groups did equally well in reducing EDE-Q subscale scores, which indicates a change in cognitions. EDE-Q subscale scores improved similarly in both our study groups, but this apparent change in cognitions did not result in improved weight-loss maintenance in the intervention group. Our results are in concordance with those of a randomised controlled trial with a follow-up period of 3 years, in which group-CBT did not improve weight-loss maintenance in obese individuals without type 2 diabetes despite improvements in cognition scores [39]. Obviously, the precise mechanisms through which CBT produces its effects are still unknown, making it unclear which measure of change in cognitions is relevant.

A recent meta-analysis showed that (group)-CBT reduces depressive symptoms in people with diabetes [40]. We found no effect of group-CBT on the HADS depression scale. However, our group-CBT was specifically designed for and aimed at maintenance of body weight loss, and not at alleviating depression.

It could be argued that individual CBT is more effective than group-CBT. In obesity research, however, group-CBT has been shown to be equally [8] or even more effective [9] than individual CBT in achieving weight loss and reducing attrition. Similarly, anxiety and obsessive compulsive disorders respond at least as well to group-CBT as to individual therapy [41, 42].

The rate of non-compliance with the group-CBT intervention in our study was 40%, which is similar to the 35–50% non-compliance reported in other CBT studies [43]. Non-compliance may have diminished the effect of the group-CBT. However, analyses restricted to the compliant group did not show an effect of group-CBT on weight at 2 years, nor at any intermediate time point. Notably, post hoc power calculations showed that we included a sufficient number of participants for the intention-to-treat as well as for the per-protocol analysis to enable relatively small differences to be detected (Table 2 of reference [13]).

The lack of an effect of group-CBT could be attributed to the psychological treatment received outside the study. The self-reported psychological consultation outside the study was similar for both groups. Alternatively, the usual care given to all our participants could have diminished the contrast between both study arms. Over the years, usual care has incorporated more and more effective elements of therapy. It is quite possible that our usual care already contains specific or non-specific treatment factors that makes it effective for weight-loss maintenance, thereby diminishing the effect of additional group-CBT. Nonetheless, we still observed a large window for improvements in maintaining weight. Whatever explains the lack of effect of group-CBT in our study, it is unlikely that our group-CBT can contribute to weight maintenance in treated obese individuals with type 2 diabetes.

Strengths of our study include the randomised controlled design and the relatively long follow-up period. Participants were referred to a single tertiary centre, and therefore our findings may not be generalisable to the entire population of overweight and obese people with type 2 diabetes. Obviously, our findings are exclusively applicable to individuals who are motivated and able to lose ≥5% of their body weight by dieting. Finally, our trial had a pragmatic design, not compensating for the increased attention received by the intervention group. Despite this increased attention, there were no differences in outcomes between the two arms, thus strengthening our conclusions.

From this high-quality randomised controlled trial, we conclude that despite increased treatment contact, group-CBT is not more effective for maintaining long-term weight loss than usual care alone in overweight and obese individuals with type 2 diabetes who were initially able to lose a clinically relevant amount of body weight on a VLCD. Future research should focus on identifying other CBT approaches in combination with other therapies to improve the long-term effectiveness of weight-loss dieting.

## Electronic supplementary material

ESM (PDF 501 kb)

## Data Availability

The datasets used and analysed during the current study are available from the corresponding author on reasonable request.

## Abbreviations

AHEAD: Action for Health in Diabetes
CBT: Cognitive behavioural therapy
Group-CBT: Cognitive behavioural group therapy
EDE-Q: Eating Disorder Examination Questionnaire
HADS: Hospital Anxiety and Depression Scale
POWER: Prevention of Weight Regain (study)
VLCD: Very low calorie diet
