# Supplementary material for: Group cognitive behavioural therapy and weight regain after diet in type 2 diabetes: results from the randomised controlled POWER trial
Source: Diabetologia. 2018 Jan 9;61(4):790–9. doi: 10.1007/s00125-017-4531-9 (PMC6448975; doi:10.1007/s00125-017-4531-9)
Supplement: Supplementary file 1 — (PDF 501 kb) [file 125_2017_4531_MOESM1_ESM.pdf]

Electronic supplementary material

ESM Table 1: Baseline characteristics of excluded vs. included participants

**Characteristic[a]**	**Excluded participants[b] (n=48)**	**Included participants (n=158)**	**p-value[c]**
Age (y, range)	50.6 (25-70)	53.7 (28-74)	0.141
Female (No. (%))	30 (62.5)	88 (55.7)	0.404
Caucasian (No. (%))	25 (52.1)	87 (55.1)	0.717
Low education (No. (%))	14 (29.2)	46 (29.1)	0.497
Years after diagnosis T2D	8.0 (2.5-13.0)	9.0 (3.0-15.0)	0.414
Weight (kg)	106.9±16.9	106.1±20.8	0.806
$HbA1_c$ (%)	8.5 (7.6-10.0)	7.6 (7.0-8.5)	0.001
$HbA1_c$ (mmol/mol)	69.5 (59.3-86.3)	60.0 (53.0-69.0)	0.001
Fasting glucose (mmol/l)	10.1 (8.3-13.0)	8.4 (6.8-10.6)	<0.001
LDL cholesterol (mmol/l)	2.6 (2.0-3.5)	2.6 (2.1-3.1)	0.706
Insulin users (No. (%))	25 (52.1)	101 (63.9)	0.456
Insulin dose among users (IU/day)	111.1±61.6	97.8±49.0	0.258
Depression score (HADS)	7.0 (4.0-11.0)	7.0 (4.0-9.0)	0.395
Anxiety score (HADS)	8.0 (4.0-11.0)	6.0 (3.0-9.0)	0.038
Quality of life (EQ5D score)	0.81 (0.69-0.84)	0.78 (0.65-0.84)	0.320
Eating disorder (EDE-Q score)	2.1±1.3	2.2±1.1	0.946
Physical activity (SQUASH score)	4200 (1440-8405)	2940 (1350-5775)	0.327

[a]Data are mean±SD, median (interquartile range) or number (%).[b]Participants were excluded when they lost <5% of body weight during the 8-week phase-in very low-calorie diet. [c]Between-group differences were analysed using a Pearson Chi-Square test, an independent samples t-test or a Mann-Whitney U test, depending on normality of the data.

T2D = type 2 diabetes; $HbA1_c$ = glycated haemoglobin; LDL = low-density lipoprotein; HADS = Hospital Anxiety and Depression Scale; EQ5D = EuroQol five-dimension questionnaire; EDE-Q = Eating Disorder Examination Questionnaire; SQUASH = Short Questionnaire to Assess Health Enhancing Physical Activity

ESM Table 2: Linear mixed model of weight course from baseline to two years of follow-up and allocation to group (Intention-to-treat)

Parameter	Estimate	95%CI	p-value
Intercept	106.769	102.327, 111.211	<0.001
Time	0.361	0.293, 0.429	<0.001
Quadratic time	-0.001	-0.002, -0.001	<0.001
Logarithmic time	-5.818	-6.400, -5.237	<0.001
Allocation	-1.274	-7.403, 4.855	0.682
Time*allocation	-0.033	-0.127, 0.061	0.491
Quadratic time*allocation	0.000	-0.001, 0.001	0.720
Logarithmic time*allocation	0.473	-0.331, 1.278	0.248
